# Erratum: Fabrication of Chitin/Poly(butylene succinate)/Chondroitin Sulfate Nanoparticles Ternary Composite Hydrogel Scaffold for Skin Tissue Engineering. *Polymers*, **2014**, *6*, 2974–2984

**DOI:** 10.3390/polym11030466

**Published:** 2019-03-11

**Authors:** S. Deepthi, C. V. Sidhy Viha, Chaochai Thitirat, Tetsuya Furuike, Hiroshi Tamura, Rangasamy Jayakumar

**Affiliations:** 1 Amrita Centre for Nanosciences and Molecular Medicine, Amrita Institute of Medical Sciences and Research Centre, Amrita Vishwa Vidyapeetham University, Kochi-682 041, India; deepthisankar@aims.amrita.edu (S.D.); sidhyvihacv@aims.amrita.edu (C.V.S.V.); 2 Faculty of Chemistry, Materials and Bioengineering, Kansai University, Osaka 564-8680, Japan; thitirat777@hotmail.com (C.T.); furuike@kansai-u.ac.jp (T.F.)

The authors wish to make a change to the published paper [1]. In the original manuscript, Figure 6B was inadvertently the same as Figure 6E. The corrected Figure 6 is presented below.

The authors apologize for any inconvenience caused. The change does not affect the scientific results. The manuscript will be updated, and the original will remain online on the article webpage https://www.mdpi.com/2073-4360/6/12/2974.

## Figures and Tables

**Figure 6:**
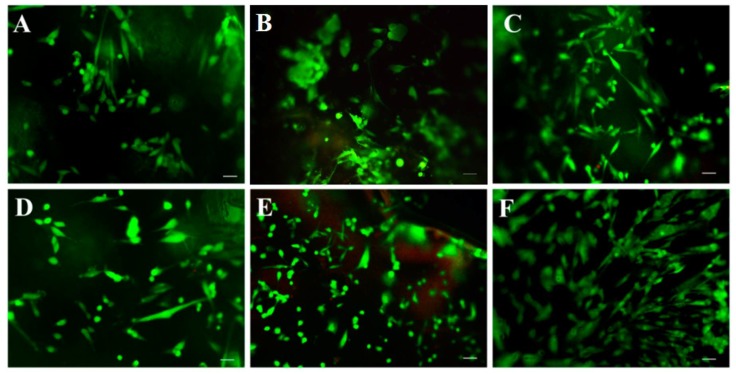
Live–dead assay of HDFs on (**A**,**D**) Chitin, (**B**,**E**) Chitin/PBS, (**C**,**F**) Chitin/PBS/CSnps after 24 h (**A**–**C**), and 48 h (**D**–**F**). Scale bar denotes 50 μm.
